# Ductility Enhancement of Sustainable Fibrous-Reinforced High-Strength Lightweight Concrete

**DOI:** 10.3390/polym14040727

**Published:** 2022-02-14

**Authors:** Md. Akter Hosen, Mahaad Issa Shammas, Sukanta Kumer Shill, Safat Al-Deen, Mohd Zamin Jumaat, Huzaifa Hashim

**Affiliations:** 1 Department of Civil and Environmental Engineering, College of Engineering, Dhofar University, P.O. Box 2509, Salalah PC 211, Oman; 2 School of Engineering, Deakin University, Waurn Ponds, VIC 3216, Australia; sukanta.shill@deakin.edu.au; 3 School of Engineering and Information Technology, The University of New South Wales, Canberra, ACT 2612, Australia; s.al-deen@adfa.edu.au; 4 Department of Civil Engineering, Faculty of Engineering, University of Malaya, Kuala Lumpur 50603, Malaysia; zamin@um.edu.my (M.Z.J.); huzaifahashim@um.edu.my (H.H.)

**Keywords:** compression ductility, displacement ductility, energy ductility, deformability, energy absorption, fibrous reinforced, lightweight concrete

## Abstract

To limit the cross-sectional size of concrete structures, high-strength, lightweight concrete is preferred for the design and construction of structural elements. However, the main drawback of high-strength, lightweight concrete is its brittleness over normal-weight concrete. The ductility of concrete is a crucial factor, which plays an important role when the concrete structures are subjected to extreme situations, such as earthquakes and wind. This study aims to improve the ductility of high-strength, lightweight concrete by incorporating steel fibers. The palm oil clinker (POC)-based, high-strength, lightweight concrete specimens reinforced with steel fibers were prepared and their ductility was systematically examined. POC was used as aggregates and supplementary cementitious materials. Steel fibers from 0–1.50% (by volume), with an increment of 0.5%, were used in the concrete mix. Compression ductility, displacement ductility and energy ductility were used as indicators to evaluate the enhancement of ductility. Moreover, the compressive strength, flexural strength, stress-strain behavior, modulus of elasticity, load-displacement characteristics, energy absorption capacity and deformability of the concrete samples were investigated. The compression ductility, displacement ductility and energy ductility indexes were found to be increased by up to 472%, 140% and 568% compared to the control specimens (concrete with 0% steel fibers), respectively. Moreover, the deformability and energy absorption capacity of the concrete were increased by up to 566% and 125%, respectively. Therefore, POC-based, high-strength, fibrous, lightweight concrete could perform better than conventional concrete under extreme loading conditions as it showed significantly higher ductility.

## 1. Introduction

Lightweight concrete (LWC) fabrication has a significant impact on modern construction industries due to its importance in infrastructural construction worldwide [1,2]. LWC is more beneficial than regular-weight concrete as it imposes significantly fewer dead loads on the load-carrying elements (beam, column and foundations) of infrastructures. Additionally, it offers more efficient sound insulation, relatively lower transportation costs of precast units and requires fewer props during construction [3,4,5]. LWC has been widely utilized in the construction of high-rise buildings, large-span bridges, sidewalks, steel structure protective layers or steel–concrete composite structures and various prefabricated concrete sections [6,7,8].

Adequate ductility for high-rise buildings in earthquake-prone areas is vigorously essential [9,10]. Moreover, the ductility of lightweight concrete is required when structures are subjected to blast loading [11,12]. Flexural and rupture strength, along with stiffness, have also been focused on in different investigations of its uses in tunnels, rigid pavements, piping and gradient covering [13,14,15]. Recently, many investigators have led experimental investigations on the properties of LWC using locally available waste materials [16,17,18,19].

A lot of waste materials are produced by the palm oil manufacturing industries in the form of oil palm shell (OPS) and POC in Malaysia [20]. Malaysia is one of the leading palm oil manufacturing countries in the world since it manufactures more than 50% of the world’s palm oil [21]. Malaysia mass-produces more than 4.0 million tons of OPS and a massive quantity of POC as waste materials every year [22]. The OPS and POC are both eco-friendly materials with a high potential as lightweight aggregates (LWA) for concrete construction industries. However, it is considered agricultural waste material. Some recently published studies revealed that OPS and POC can be utilized as LWA for fabricating LWC, targeting structural application [23,24].

Mohammed et al. [25] reported that POC-based concrete beams met the deflection conditions under the BS8110 code when a reinforcement ratio of less than 0.524% was used. The experimental investigation was conducted comprising eight under-reinforced beams fabricated with differing reinforcing steel ratios ranging from 0.34% to 2.21%. The outcomes exhibited the deflection, cracking characteristic and ductility indexes. Additionally, the ductility index decreased with the increasing reinforcement ratio in the steel-fiber-reinforced, POC-based concrete beams. Shafigh et al. [26] investigated the influence of steel fiber on the mechanical properties of OPS lightweight concrete. The OPS concrete comprised volume fractions of steel fiber of 0%, 0.25%, 0.5%, 0.75% and 1%. The mixtures of the OPS lightweight concrete had a superplasticizer of 0.7% by cement weight. The experimental outcomes yielded that the amount of steel fibers significantly influenced the slump, density, compressive strength under several curing conditions, splitting tensile strength and flexural strength, except for the modulus of elasticity, compared to the control specimen. Medeiros et al. [27] mentioned that the inclusion of fibers in concrete influences its strength and modulus of elasticity but is reliant on the volume fractions of fibers used in the mixture. The distinct loading frequencies of 1/16 Hz, 1/4 Hz, 1 Hz and 4 Hz were applied for experimental investigation. The highest level of stress was 85% of its compressive strength, which was employed on the sample, and the ratio of stress was restrained constantly as 0.3. Fibers might have enhanced the assimilated air content, which deleteriously impacted the compacting characteristics. Teo et al. [28] stated that LWC beams with reinforcement ratios of 0.52% and 3.90% fulfilled the maximum acceptable deflection limits according to the BS code [29] at service loads levels. A sum of six under-reinforced beams with a cross-section of 150 mm × 225 mm with different reinforcing steel ratios was constructed and examined. However, at a reinforcement ratio of 1.13%, the specimen’s deflection exceeded its maximum limit. 

Therefore, the prominent objective of the present study is to enhance the ductility performance of POC-based, high-strength, lightweight concrete comprising steel fibers of different volume fractions. The energy absorption capacity, ductility (compression, displacement and energy) and deformability properties are used as indicators to assess the ductility of the POC-based concrete with steel fibers.

## 2. Experimental Program

### 2.1. Materials

#### 2.1.1. Cement

The locally available (Tasek cement [30]) ordinary Portland cement (OPC) type I, based on ASTM [31], was utilized for manufacturing the fibrous, reinforced, high-strength, lightweight concrete. Table 1 reveals the chemical arrangement of OPC [32]. The specific gravity, bulk density and Blaine specific surface area of OPC were about 3.14, 1657 kg/m^3^ and 351 m^2^/kg, respectively.

#### 2.1.2. POC Powder

POC powder was utilized as supplementary bonding materials for fabricating high-strength, lightweight concrete [32]. The POC powder was manufactured by grinding the POC particles (5 mm size) with a stone grinder machine in the concrete laboratory. The POC powder was comprised of constituents of amorphous and crystalline solid elements. The chemical configuration of POC powder is revealed in Table 2 [32].

#### 2.1.3. Superplasticizer

A superplasticizer was applied in the mixes to reduce the water quantity and enhance the workability during the production of fresh, fibrous, reinforced, high-strength, lightweight concrete. The polycarboxylate ether (PCE)-based Sika ViscoCrete^®^-2199 [33] was utilized as a superplasticizer.

#### 2.1.4. Water

Drinkable tap water was applied for manufacturing and curing the fibrous, reinforced, high-strength, lightweight concrete. The alkalinity and pH values of the water were 135 mg/L and 6.50, respectively.

#### 2.1.5. Aggregates

Artificial mining sand was used as a fine aggregate in this study. The sand was graded using the mechanical sieve in the laboratory and was passed through a 4.75 mm sieve and retrained 300 μm sieve. The water absorption capacity, specific gravity and fineness modulus of the sand were 1.18%, 2.65 and 2.88, respectively.

**Figure 1:**
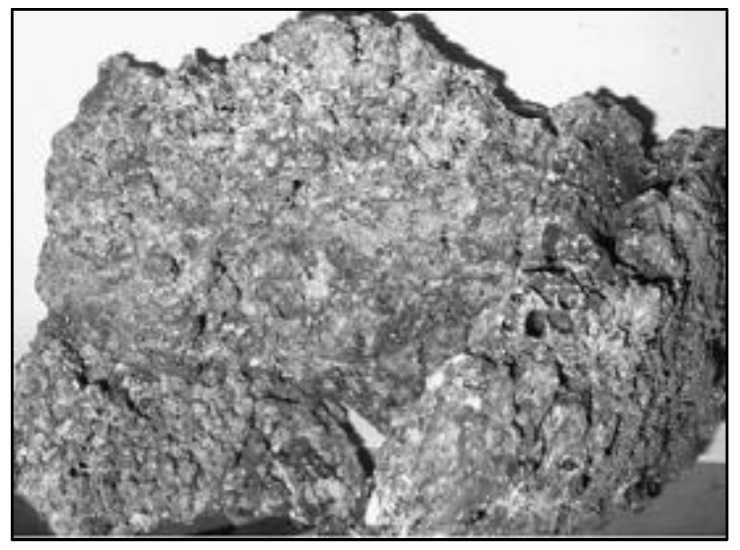
POC boulder before crushing.

The POC waste chunk was utilized as a lightweight coarse aggregate for constructing high-strength, lightweight concrete [32]. The raw POC boulder was accumulated from palm oil manufacturing industries, as demonstrated in Figure 1.

**Figure 2:**
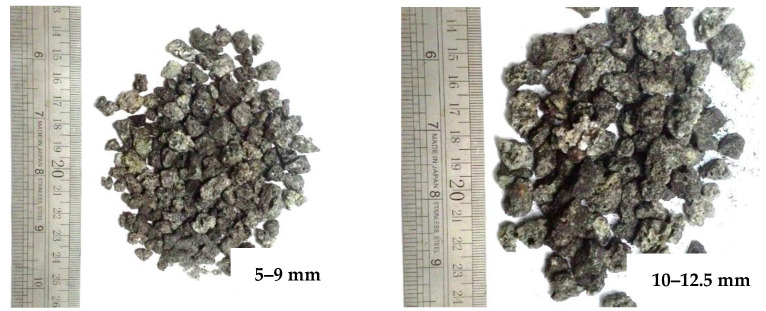
Waste POC coarse aggregate.

The boulder POC was materialized from the ignition of palm shells and fibers to produce energy. That energy was used to manufacture oil. The POC boulder was crushed to a specified size of 5 mm to 12.50 mm with a stone grinder machine and utilized as lightweight aggregate, as presented in Figure 2.

#### 2.1.6. Steel Fibers

**Figure 3:**
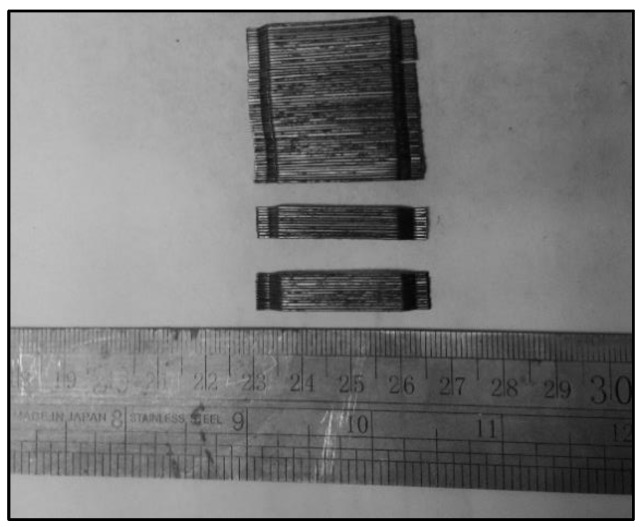
Glued, hooked-end steel fibers.

The glued, hooked-end, bundle-type steel fibers were employed in the mixes for producing fibrous, reinforced, high-strength, lightweight concrete [34]. Figure 3 reveals the hooked-end steel fibers. The properties of the steel fibers were provided by the manufacturer and are demonstrated in Table 3. The quantity of the fibers varied from 0 to 1.50%, as shown in Table 4, for assessing the ductile performance of the fibrous, lightweight concrete. 

### 2.2. Mix Procedure and Specimens Preparation

A total of four mixes were cast with various fractions of steel fibers: 0%, 0.50%, 1.0% and 1.5%. Each of the mixes was prepared with a fixed amount of cement, POC powder, fine aggregate, POC aggregate, water and SP. The mix proportions for fabricating the fibrous, reinforced, high-strength, lightweight concrete are presented in Table 4. The mixture comprising 0% of steel fiber was considered as a reference mix and labelled as HC-F0. The remaining mixtures were comprised of 0.50%, 1.0% and 1.5% steel fibers and branded as HC-F.50, HC-F1.0 and HC-F1.5, respectively. The waste POC powder was applied as a supplementary bonding material, along with the required amount of cement, to reduce the dependency on the cement and ensure sustainability by declining the cement quantity. The POC powder was applied at 15% by weight of the cement in this study. The local, artificial mining sand and POC aggregate were utilized in the mixtures of the fibrous, lightweight concrete as fine and coarse aggregates, respectively.

For fabricating the mixture of the fresh, lightweight concrete, at first, the POC aggregate and local, artificial sand were mixed and blended in the mixer machine for approximately 2 min. After that, the cement and supplementary bonding materials (POC powder) were added to the mixture and mixed for approximately 3 more minutes. Seventy percent of the essential water was added into the mixture and blended for 5 more minutes. The remaining 30% of essential water and SP were added into the mixture, and mixing was prolonged for 5 more minutes. Finally, the steel fibers were added into the mixture as a reinforcing agent, and the mixing was resumed for a further 5 min. The workability of the fresh, fibrous, lightweight concrete was ensured by performing a slump test before the casting of the specimens. The fresh concrete was cast in 100 mm cubical, 100 mm × 100 mm × 500 mm prism and 100 mm × 200 mm cylinder molds to evaluate the compressive strength, flexural strength and tensile strength, respectively. The cylindrical specimens were also used to assess the stress–strain behavior and modulus of elasticity of the lightweight concrete. All specimens were de-molded after 24 h of casting and kept in the potable water tank for curing reasons until the testing of the specimens.

### 2.3. Test Methods of the Specimens

#### 2.3.1. Compressive Strength Test

The compressive strength tests of the cube specimens were conducted as per BS EN 12390-3 [35] using an automatic compression (ELE) testing machine. The cube specimens (100 mm × 100 mm × 100 mm) were tested after a curing period of 28 days. Three specimens were tested for each category of the mixture, and the average values of the compressive strength were documented.

#### 2.3.2. Flexural Strength Test

**Figure 4:**
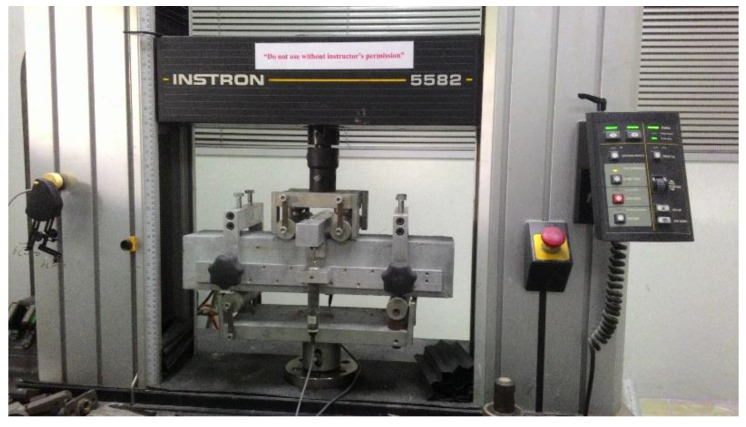
Experimental setup.

The flexural strength tests were carried out using 100 mm × 100 mm × 500 mm prism specimens following BS EN 12390-5 [36]. The specimens were tested under four-point bending until failure. The experimental setup is demonstrated in Figure 4. The tests were executed at a constant displacement control rate of 0.00083 mm/s. The applied load on the specimens was captured by the Universal Instron machine (model no. 5582) throughout the experiments. A linear variable displacement transducer (LVDT) was utilized for measuring the deflection of the specimens, and it was recorded in the TDS 530 data logger during the tests.

#### 2.3.3. Modulus of Elasticity (MOE) Test

**Figure 5:**
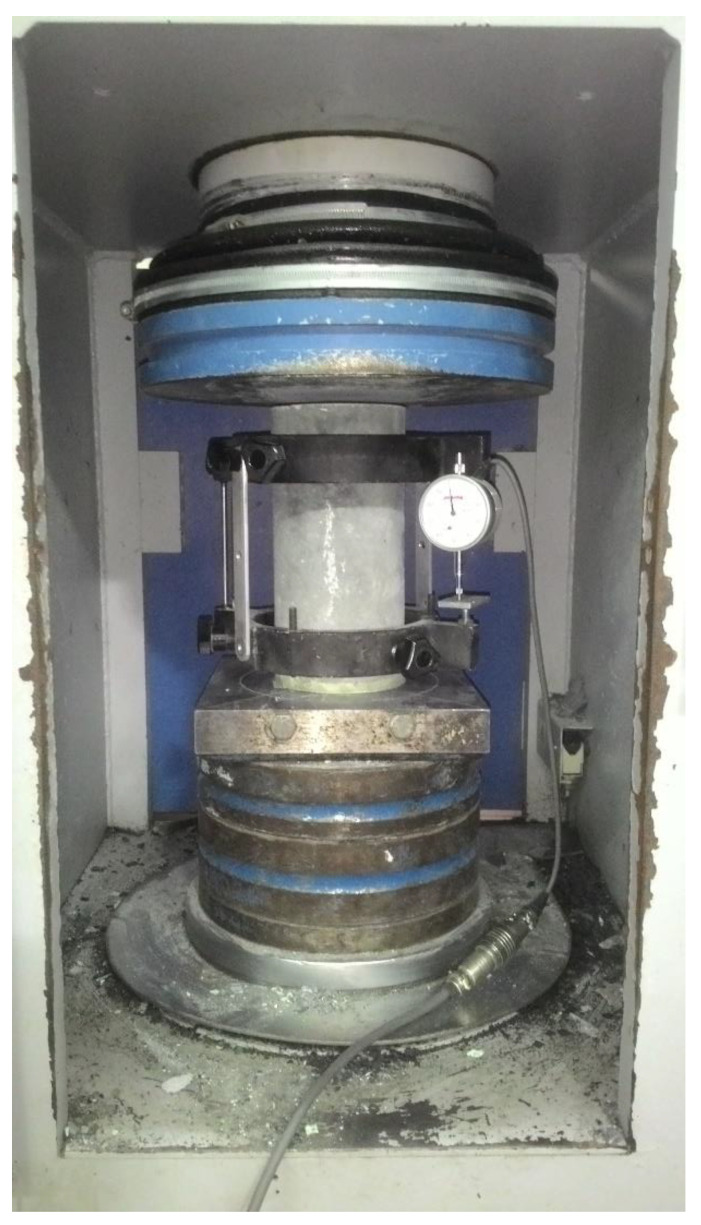
Experimental setup for MOE test.

The MOE was computed from the stress–strain relationship of the fibrous, reinforced high-strength, lightweight concrete following ASTM C469 [37]. The stress–strain was assessed from the cylindrical specimens (100 mm diameter × 200 mm height) tested under compressive load using an ELE compression machine, as shown in Figure 5. The compressive load was captured by the ELE machine, and the corresponding longitudinal deformation was measured using a compression extensometer (model no. C131N1). Both the ELE machine and extensometer were connected to the TDS 530 data logger, and data were automatically recorded in the data logger during the test.

## 3. Experimental Outcomes and Discussions

### 3.1. Compressive Strength

**Figure 6:**
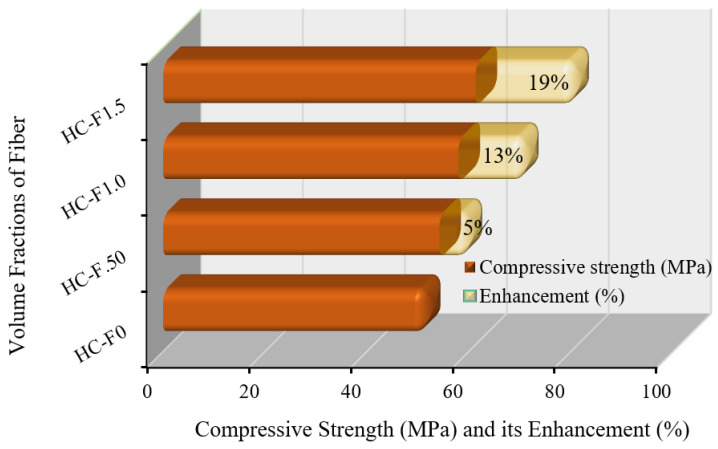
Compressive strength enhanced by the steel fibers.

According to the experimental outcomes, sustainable, POC-comprising, fibrous, reinforced, high-strength, lightweight concrete exhibited a compressive strength ranging from 54.16 MPa to 61.26 MPa with a volume fraction of fiber from 0.50% to 1.50%. The standard deviation values lay between 0.32 and 1.09. Figure 6 demonstrates that the highest compressive strength was enhanced by up to 19% compared with the reference specimen. This compressive strength of the fibrous, reinforced, high-strength, lightweight concrete differed due to the strength of the lightweight aggregates and the bond among the cementitious paste, aggregates and steel fibers [38]. Since POC aggregates are very porous compared with regular crushed stone aggregates [39], the achievement of the high compressive strength of this sustainable concrete was contributed by the mortar and strong bonding matrix between the mortar and steel fibers [40].

### 3.2. Flexural Strength

**Figure 7:**
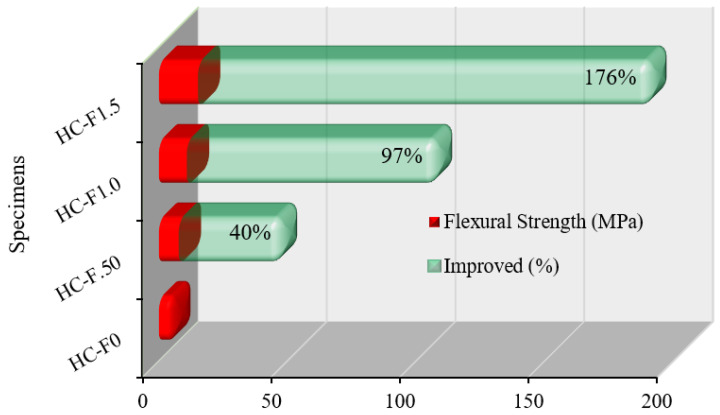
Flexural strength enhancement of POC lightweight concrete by steel fibers.

Figure 7 exhibits the flexural strength and its improvement for POC, fibrous, reinforced, high-strength, lightweight concrete specimens after 28 days of curing. The flexural strengths were measured for all mixes by implementing four-point bending loading circumstances. The flexural strength of the sustainable, POC-encompassing, fibrous, high-strength, lightweight concrete was revealed to be 5.5 MPa, 7.69 MPa, 10.83 MPa and 15.17 MPa for HC-F0, HC-F.50, HC-F1.0 and HC-F1.5, respectively. The standard deviation values lay between 0.78 and 1.44. As compared to HC-F0, the corresponding flexural strength improvement was found to be 40%, 97% and 176%. The higher volume fraction (1.50%) of steel fibers significantly improved the flexural strength. This improvement was due to the robust interlocking bond mechanism between the POC concrete and steel fibers. However, the inclusion of a smaller volume of steel fiber did not influence the flexural strength of the lightweight concrete, though it substantially enhanced the ductile performance [41]. Based on ASTM C330/C330M-17a [42], the required flexural strength was 2.13 MPa for lightweight aggregate concrete. Hence, sustainable, fibrous, reinforced, high-strength, lightweight concrete might be useful for structural application in the construction industry.

The oil-palm-shell, lightweight concrete with 1.0% steel fibers had increased flexural strength by up to 31% compared with the control specimen [26]. Conversely, the normal, high-strength concrete had an enhanced flexural strength of 81% compared with the control specimen, incorporating a volume fraction of steel of 1.50% [43]. The self-compacting geopolymer concrete with a steel fiber contents of 1% had 28% greater flexural strength over the control specimen [44].

### 3.3. Stress–Strain Behavior

**Figure 8:**
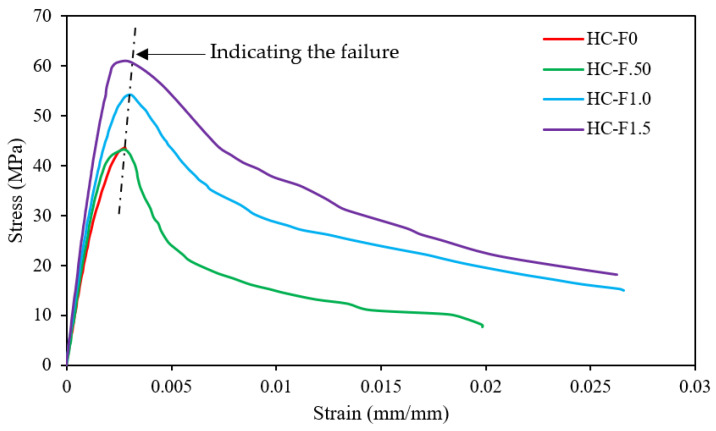
Stress–strain characteristics of the fibrous reinforced lightweight concrete.

The stress–strain relationships for POC-containing, fibrous, reinforced, high-strength, lightweight concrete are presented in Figure 8. The figure denotes that the efficiency of the ultimate stress capacity improved with increasing steel fibers content in the POC, fibrous, reinforced, high-strength, lightweight concrete. It can also be perceived that increasing steel fibers content in the POC concrete reduced the gradient of the stress versus strain graph (steeper) in the pre-peak stage due to the decrease in strain values caused by the increased fiber contents. In the stress–strain graph, it can be appreciated that the strain values at the rupture point of the specimens were found at 0.002812, 0.019819667, 0.026578333 and 0.026242 for HC-F0, HC-F.50, HC-F1.0 and HC-F1.5, respectively, with only the reference specimen value being very close to the maximum stress. The reference specimen demonstrated a brittle failure, whereas the other steel-fibers-comprising specimens confirmed ductile failure.

**Figure 9:**
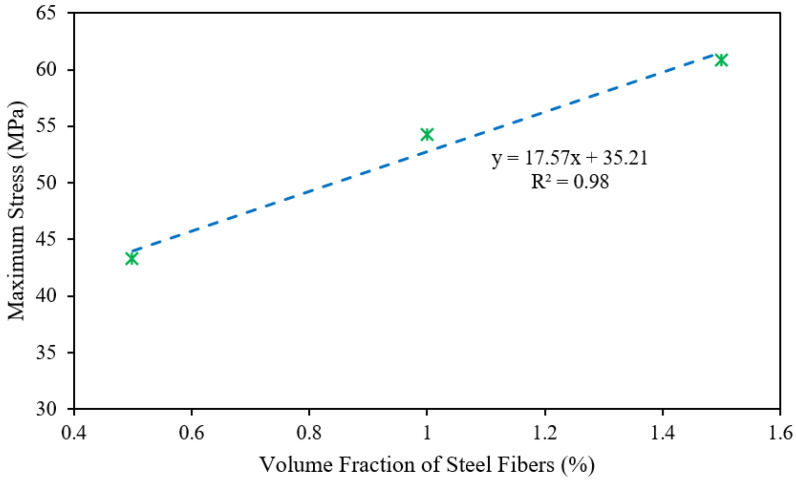
Relationship between the maximum stress and steel fiber content.

Figure 9 shows the relationship between the maximum stress and steel fibers content for POC-incorporating, fibrous, reinforced, high-strength, lightweight concrete specimens. A relationship with a strong correlation (R^2^) was anticipated for fibrous, reinforced, high-strength, lightweight concrete. The anticipated equation for high-strength, lightweight concrete is specified by:

(1) $$\delta_{max}=17.57V_{f}+35.21$$

where $\delta_{max}$ is the maximum stress of the fibrous, reinforced, high-strength, lightweight concrete, and *V_f_* is the volume fraction of the steel fibers.

Balendran et al. [45] reported that normal-weight concrete causes abrupt failure after the ultimate strength. Therefore, steel fibers increase the strain capability of POC-containing, high-strength, lightweight concrete. Similar characteristics were identified for steel-fibers-comprising, natural, lightweight aggregate concrete [46].

### 3.4. Modulus of Elasticity

The modulus of elasticity (MOE) is of major significance for the strength of concrete for structural application. In addition, ACI 318-19 [47] specified that the MOE of the concrete relies on its density and compressive strength. In the circumstance of POC-comprising, fibrous, reinforced, high-strength, lightweight concrete, the increase in the modulus of elasticity (up to 40%) appeared to be influenced substantially by the volume fraction of steel fibers. 

**Figure 10:**
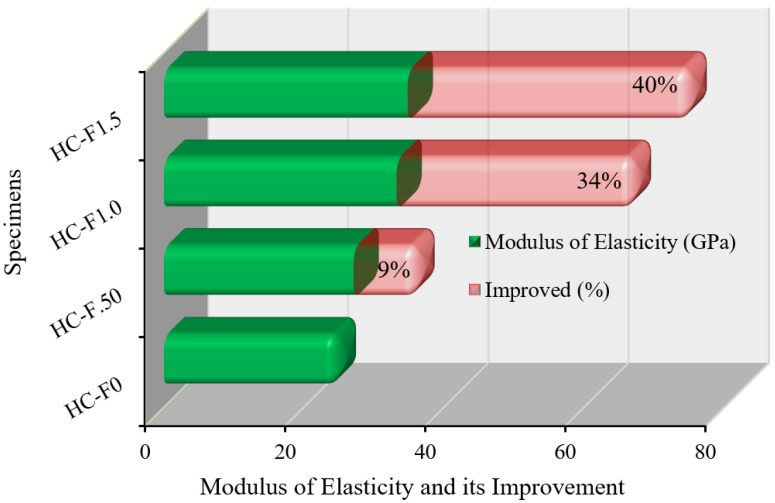
Modulus of elasticity of the fibrous reinforced lightweight concrete.

The MOE and its improvement in the sustainable, fibrous, reinforced, high-strength, lightweight concrete specimens are revealed in Figure 10. The values of the static MOE of HC-F0, HC-F.50, HC-F1.0 and HC-F1.5 were 24.83 GPa, 27.06 GPa, 33.20 GPa and 34.78 GPa, respectively. These assessments revealed that the addition of steel fibers to the sustainable, POC, high-strength, lightweight concrete significantly improved its MOE. The standard deviation values lay between 1.68 and 2.47. Steel fibers enhanced the stiffness and reduced the displacement at pre-peak phase of the POC-containing, high-strength, lightweight concrete, although FIB reported [48] that the MOE of structural, lightweight concrete was between 10 GPa and 24 GPa.

The MOE of the oil-palm-shell-with-steel-fiber, lightweight concrete was lower than that of the reference specimen (HC-F0). The highest MOE value was 16.1 GPa for an oil-palm-shell concrete with 0.25% steel fibers, whereas the cement content was 500 kg/m^3^. Furthermore, the outcomes presented that gradually increasing the steel fibers content in the oil-palm-shell concrete reduced the MOE [26].

### 3.5. Load-Displacement Behavior

**Figure 11:**
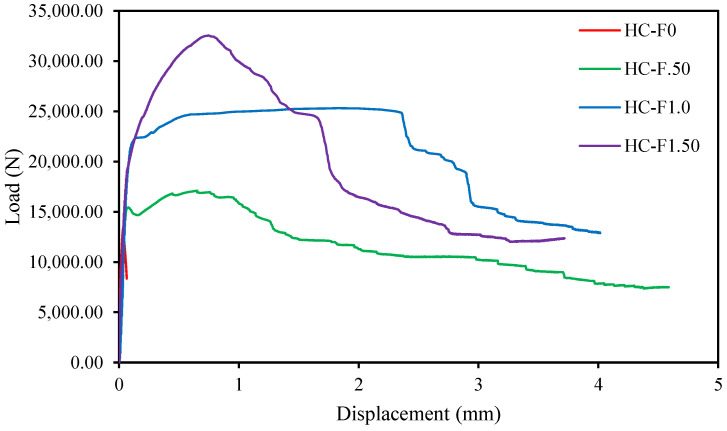
Load-displacement behavior of the fibrous reinforced lightweight concrete.

The load-displacement graph is very essential for revealing the flexural characteristics of the structural members, which signify the serviceability of the concrete members. The load-versus-displacement correlation is influenced by the first crack load, yield load, ultimate load and stiffness constraints of the structural members. Figure 11 exhibits the load-displacement graphs of the POC-comprising, fibrous, reinforced, high-strength, lightweight concrete specimens. The load-displacement graphs reveal an almost identical trend in the pre-peak phase for all specimens [49]. Hence, the plastic phase was more dominant than the elastic phase of the fibrous, reinforced, high-strength, lightweight concrete specimens. While the reference specimen graph shows elastic performance at the pre-peak phase, there is an abrupt drop of the gradient after the peak due to the brittle failure. Therefore, the flexural strength of the POC-containing, high-strength, lightweight concrete without steel fibers specimen was too low, and it would be always ignored in the design of concrete members.

The pre-peak phases (two distinct) of the fibrous, reinforced, high-strength, lightweight concrete specimens’ graphs can be described as follows: the first phase of the displacement graphs can be characterized as the elastic linear where there was no crack initiated in the tension face, and the full flexural stiffness and rigidity was stated. The second phase of the graphs represents the commencement of flexural cracks and a decrease in the gradient of the graphs. The flexural stiffness of the specimens gradually reduced with the increase of the applied load. The post-peak also displayed two distinct phases, except for the reference specimen. In the third phase, the gradient of the fibrous, reinforced specimens exhibited less slope compared with the previous phase. The flexural stiffness of the specimens depended on the tensile strength of the steel fibers due to all the stress transferred to the fibers at this phase. Thus, this phase accumulated more energy compared to any phases of the specimens, which confirmed the ductile failure of the fibrous, reinforced, high-strength, lightweight concrete specimens. The HC-F1.0 specimen (1% steel fibers) was revealed to be more ductile compared with the HC-F0, HC-F.50 and HC-F1.5 (0%, 0.50% and 1.50% steel fibers) specimens due to proper bridging between the steel fibers and the POC-containing, lightweight concrete and the more homogeneous nature of the mixture. In the final phase, the POC, lightweight concrete was critically damaged, and the steel fibers lost their tensile strength gradually then fractured and ensured rupture failure of the specimens.

### 3.6. Energy Absorption Capacity

The energy absorption capacity (EAC) of the specimens is expressed as the energy stored by the unit cross-sectional area at any displacement terminal point [50]. The EAC of the specimens was computed by employing the area under the load vs. displacement plots up to the rupture of the specimens based on Figure 11 [51].

Figure 11 exhibits that the POC-comprising, high-strength, lightweight concrete specimen (HC-F0) without fibers unexpectedly failed when the displacement exceeded its ultimate loading point [52]. On the other hand, POC, high-strength, lightweight concrete specimens with steel fibers continued to support greater loads (HC-F.50, HC-F1.0 and HC-F1.5) than the lightweight concrete without steel fibers and demonstrated larger displacement. The assessment of the ruptured specimens revealed that the failure appeared after the fracture of the steel fibers due to the crack-bridging influence between the steel fibers and the mortar matrix. Hence, the steel fibers were prolonged to elongate until the splitting. This expressed that the specimens with steel fibers had higher robustness over the specimen without steel fibers. The robustness of the specimens signified the energy absorption capability [53].

**Figure 12:**
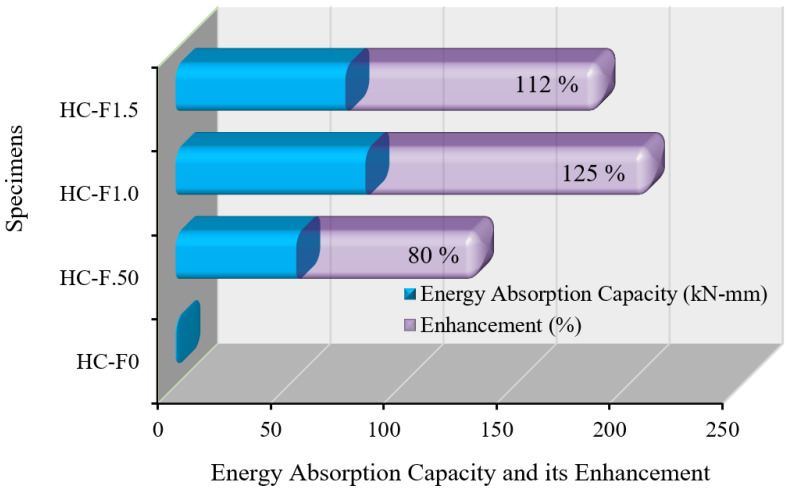
EAC of the POC-containing fibrous reinforced lightweight concrete.

The EAC and its enhancement of the sustainable POC, fibrous, reinforced, lightweight concrete specimens is revealed in Figure 12. The percentage increase of the EAC of specimens with steel fibers was 80%, 125% and 112% over that of the specimen without steel fibers. The figure demonstrates that increasing the amount of wire steel fibers from 0% to 1.5% in the POC-containing, lightweight concrete enhanced EAC from 0% to 125%, respectively. Therefore, the HC-F1.0 specimen (1.0%) was more efficient compared with volume fractions (0.5% and 1.5%) due to the post-peak characteristics of the displacement.

### 3.7. Ductility

Ductility can be defined as the material competence to experience an inelastic distortion without dropping its strength capability. It is a crucial aspect in the design of concrete infrastructures [50,51,54]. One of the fundamental intentions of adding steel fibers to the POC-comprising, high-strength, lightweight concrete was to enhance its ductility and make it more worthy for application in infrastructures subjected to fatigue and earthquake loadings. In this study, three types of ductility, i.e., compression, displacement and energy, were assessed to check the effectiveness of the POC-comprising, high-strength, lightweight concrete.

#### 3.7.1. Compression Ductility

**Figure 13:**
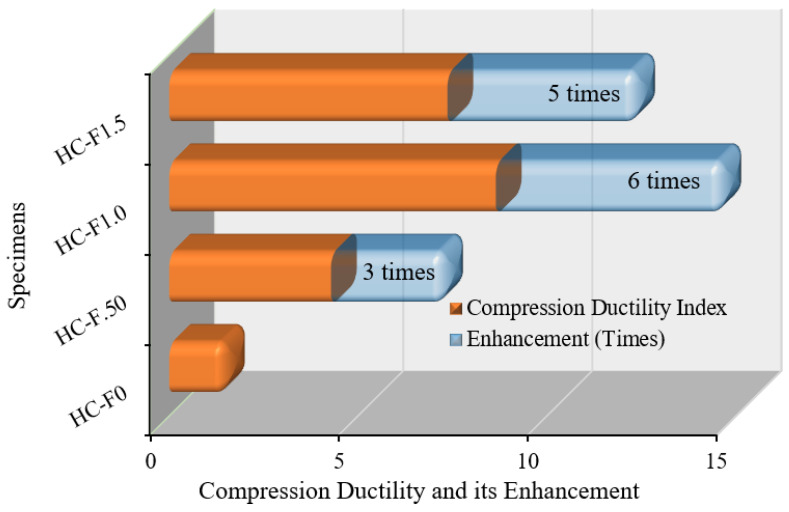
Compression ductility of the POC fibrous reinforced lightweight concrete.

In this study, the compression ductility was quantified from the stress–strain graphs (Figure 8). The compression ductility index is the ratio of the area under the stress–strain diagram at the failure at the ultimate load [55]. The compression ductility index of the POC-containing, fibrous, reinforced, high-strength, lightweight concrete specimens is demonstrated in Figure 13. The steel fibers contents in the POC-incorporating, high-strength, lightweight concrete significantly enhanced compression ductility. The volume fraction of fibers 0.50%, 1.0% and 1.50% increased the compression ductility index by 3, 6 and 5 times, respectively, compared with the reference specimen. The superior ductility index specified that the structural elements are proficient in experiencing large displacement without failure [56,57].

#### 3.7.2. Displacement Ductility

The displacement ductility index of the POC-encompassing, fibrous, reinforced, high-strength, lightweight concrete specimens was assessed from the load versus displacement graphs (Figure 11) [58]. The displacement ductility index of the lightweight concrete specimens is defined as the ratio of displacement at ultimate load to yield load [59].

**Figure 14:**
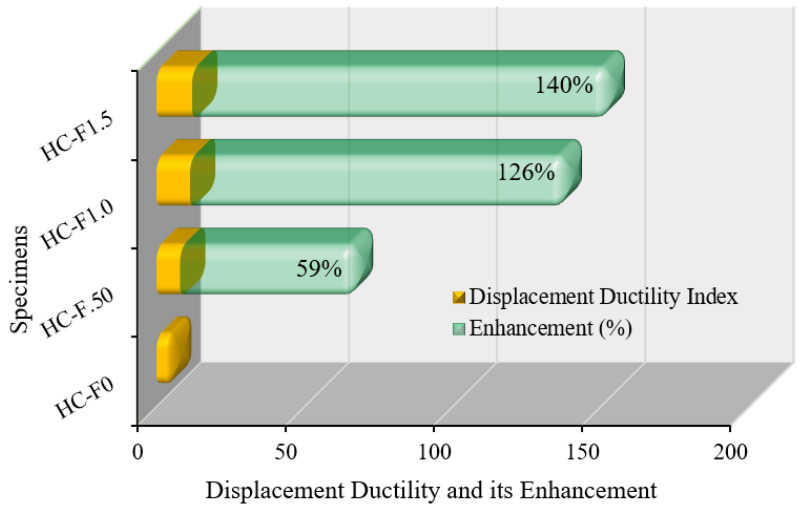
Displacement ductility of the POC fibrous reinforced lightweight concrete.

The ductility and its enhancement of the fibrous, reinforced, high-strength, lightweight concrete specimens are graphically demonstrated in Figure 14. The displacement ductility index of the POC, lightweight concrete specimens with steel fiber indicated significant enhancement of up to 140% compared with the reference specimen. Therefore, the POC-containing, high-strength, lightweight concrete specimens using steel fibers were very efficient in terms of displacement ductility.

#### 3.7.3. Energy Ductility

**Figure 15:**
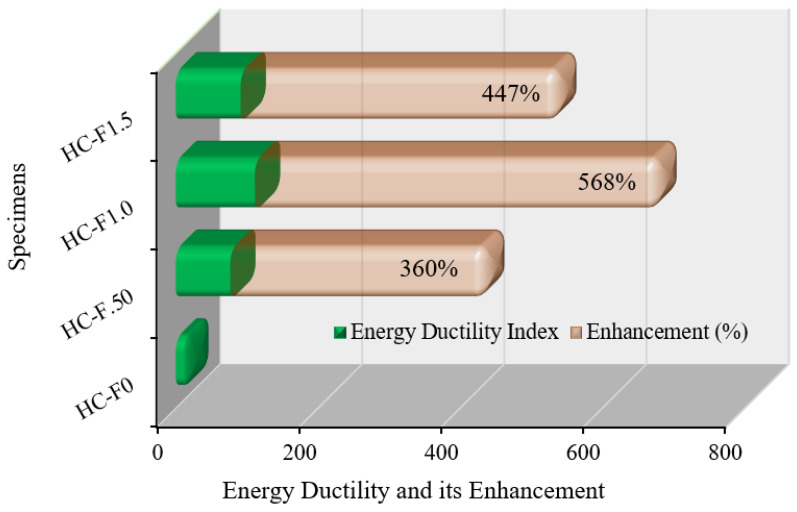
Energy ductility of the POC fibrous reinforced lightweight concrete.

The energy ductility index of the POC-encompassing, fibrous, reinforced, high-strength, lightweight concrete is expressed as the ratio of the energy at the failure to the energy at the first yield of the specimen [60]. Figure 15 specifies how the addition of steel fibers to the reinforced, high-strength, lightweight concrete enhanced the energy ductility index. The volume fraction of steel fibers remarkably increased the energy ductility of the specimens compared with the reference specimen. The energy ductility improvement for HC-F.50, HC-F1.0 and HC-F1.5 was 360%, 568% and 447%, respectively, over the reference specimen. The energy ductility improvement of the HC-F1.0 specimen was higher than the HC-F1.5 specimen, since HC-F1.5 experienced a non-homogeneous bonding matrix with steel fibers; hence, the specimen did not achieve more ductility. Therefore, the overall studies confirmed that the POC-encompassing, high-strength, lightweight concrete with steel fibers approach extensively enhances the energy ductility.

### 3.8. Deformability

**Figure 16:**
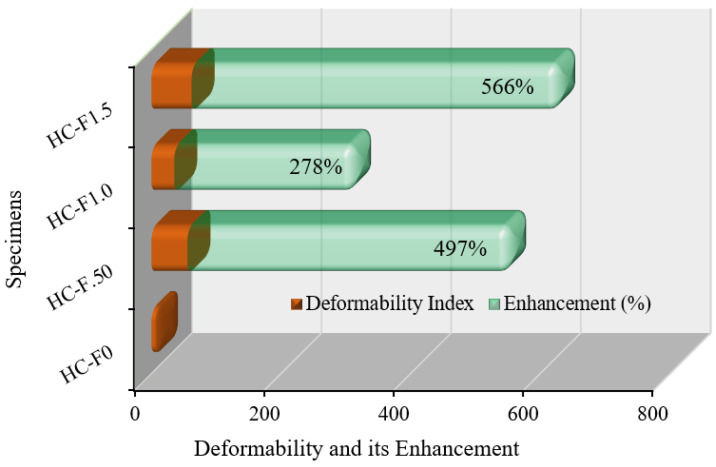
Deformability of the POC fibrous reinforced lightweight concrete.

The deformability index of the POC-incorporating, fibrous, reinforced, high-strength, lightweight concrete is expressed as the ratio of the displacement at the failure to the displacement at the yield [61,62]. Figure 16 represents the deformability index and its enhancement of the fibrous, reinforced, high-strength, lightweight concrete specimens. The deformability index of the fibrous, reinforced, high-strength, lightweight concrete specimens was substantially enhanced compared to the reference specimen. This improvement for HC-F.50, HC-F1.0 and HC-F1.5 specimens was 497%, 278% and 566%, respectively, over the reference specimen. This outcome implies that POC-including, fibrous, reinforced, high-strength, lightweight concrete has the capability to meet the deformability demands of fiber-reinforced, lightweight concrete structures.

## 4. Conclusions

This study reveals improved ductility behavior and mechanical properties of palm oil clinker (POC)-based, high-strength, lightweight concrete incorporating hook-end steel fibers (0–1.5% by volume, with an increment of 0.5%). Based on the experimental results, the following conclusions can be drawn:

(1) The compression ductility of the POC-based, high-strength, lightweight concrete with 1.0% steel fibers was six times higher compared to the control (concrete specimen with 0% steel fibers). This enhancement in ductility will significantly improve the performance of high-strength, lightweight concrete structures under earthquake and wind loading.
(2) The addition of steel fibers to the POC-based, high-strength, lightweight concrete triggered an increase in displacement ductility of up to approximately 140% over the control specimen. Moreover, the energy ductility of the POC-based, high-strength, lightweight concrete was increased by up to 568% compared to that of the control specimen.
(3) The deformability of the concrete corresponding to the peak stress increased by up to 566% owing to the addition of the steel fibers. The enhancement of the deformability of the high-strength, lightweight concrete causes a ductile failure of the concrete structures under extreme conditions.
(4) Although the 28-day compressive strength of the POC-based, high-strength, lightweight concrete increased by up to 19%, the flexural strength increased from 5.5 MPa to 15.17 MPa. This significant improvement of the flexural strength of the POC-based, high-strength, lightweight concrete results in a substantial increase in the cracking moment capacity of the structural elements.
(5) The modulus of elasticity and the energy absorption capacity of the POC-based, high-strength, lightweight concrete increased by up to 40% and 125%, respectively.
(6) Overall, the ductility and mechanical properties of the POC-based, high-strength, lightweight concrete were substantially enhanced because of the addition of hook-end steel fibers.

## Figures and Tables

**Table 1:** Chemical arrangement of OPC.

**OPC Type I**	**Chemical Arrangement (%)**						
	**CaO**	**SiO_2_**	**Al_2_O_3_**	**Fe_2_O_3_**	**MgO**	**SO_3_**	**Others**
	64	20	5	3	3	3	2

**Table 2:** Chemical configuration of POC powder.

**POC Powder**	**Chemical Configuration (%)**							
	**SiO_2_**	**K_2_O**	**Fe_2_O_3_**	**CaO**	**Al_2_O_3_**	**P_2_O_5_**	**MgO**	**Others**
	60	15	7	6	4	4	3	1

**Table 3:** Descriptions of steel fibers.

**Steel Fiber Type**	**Length, L (mm)**	**Diameter, D (mm)**	**L/D**	**Tensile Strength (MPa)**	**Modulus of Elasticity (GPa)**	**Poisson’s Ratio**	**Density (kg/m^3^)**
Hooked End	35	0.54	65	1130	200	0.28	7840

**Table 4:** Mix proportion for fibrous, reinforced, lightweight concrete [32].

Mix Category	Steel Fiber (%)	Cement	POC Powder	Fine Aggregate	POC Aggregate	Water	SP
		kg/m^3^					
HC-F0	0	420	63	765	545	189	8
HC-F.50	0.50						
HC-F1.0	1.00						
HC-F1.5	1.50						

SP—superplasticizer.

## Data Availability

The data presented in this study are available on request from the corresponding author.

## References

[1] Tayeh B.A., Zeyad A.M., Agwa I.S., Amin M. (2021). Effect of elevated temperatures on mechanical properties of lightweight geopolymer concrete. Case Stud. Constr. Mater..

[2] Graziano S.F., Zanelli C., Molinari C., de Gennaro B., Giovinco G., Correggia C., Cappelletti P., Dondi M. (2022). Use of screen glass and polishing sludge in waste-based expanded aggregates for resource-saving lightweight concrete. J. Clean. Prod..

[3] Chandra S., Berntsson L. (2002). Lightweight Aggregate Concrete.

[4] Newman J., Owens P. (2003). Properties of lightweight concrete. Adv. Concr. Technol..

[5] Clarke J.L. (1993). Structural Lightweight Aggregate Concrete.

[6] Kurpińska M., Ferenc T. (2020). Experimental and numerical investigation of mechanical properties of lightweight concretes (LWCs) with various aggregates. Materials.

[7] Wu X., Wang S., Yang J., Zhao J., Chang X. (2021). Damage characteristics and constitutive model of lightweight shale ceramsite concrete under static-dynamic loading. Eng. Fract. Mech..

[8] Dulsang N., Kasemsiri P., Posi P., Hiziroglu S., Chindaprasirt P. (2016). Characterization of an environment friendly lightweight concrete containing ethyl vinyl acetate waste. Mater. Des..

[9] Wang H., Belarbi A. (2011). Ductility characteristics of fiber-reinforced-concrete beams reinforced with FRP rebars. Constr. Build. Mater..

[10] Qin H. (2022). Decision-making under uncertainty for buildings exposed to environmental hazards. J. Saf. Sci. Resil..

[11] Taranath B.S. (2016). Tall Building Design: Steel, Concrete, and Composite Systems.

[12] Elveli B.S., Iddberg M.B., Børvik T., Aune V. (2022). On the strength–ductility trade-off in thin blast-loaded steel plates with and without initial defects—An experimental study. Thin Walled Struct..

[13] Kim S., Usman M., Park C., Hanif A. (2021). Durability of slag waste incorporated steel fiber-reinforced concrete in marine environment. J. Build. Eng..

[14] Bheel N. (2021). Basalt fibre-reinforced concrete: Review of fresh and mechanical properties. J. Build. Pathol. Rehabil..

[15] Alabduljabbar H., Mohammadhosseini H., Tahir M.M., Alyousef R. (2021). Green and sustainable concrete production using carpet fibers waste and palm oil fuel ash. Mater. Today Proc..

[16] Khan S., Yap S.P., Tan C.G., Ganasan R., Sherif M.M., El-Shafie A. (2021). Torsional Crack Localization in Palm Oil Clinker Concrete Using Acoustic Emission Method. Materials.

[17] Jagaba A.H., Kutty S.R.M., Hayder G., Baloo L., Noor A., Yaro N.S.A., Saeed A.A.H., Lawal I.M., Birniwa A.H., Usman A.K. (2021). A systematic literature review on waste-to-resource potential of palm oil clinker for sustainable engineering and environmental applications. Materials.

[18] Shafigh P., Aslam M., Yap S.P. (2021). Shear behaviour of lightweight aggregate concrete beams using palm-oil by-products as coarse aggregate. Struct. Eng. Mech..

[19] Hasan K., Yahaya F., Karim A., Othman R. (2021). Investigation on the properties of mortar containing palm oil fuel ash and seashell powder as partial cement replacement. Construction.

[20] Achaw O.-W., Danso-Boateng E. (2021). Manufacture of crude palm oil and refined palm oil. Chemical and Process Industries.

[21] Abbasi A., Yahya W.Z.N., Nasef M.M., Moniruzzaman M., Ghumman A.S.M. (2021). Copolymerization of palm oil with sulfur using inverse vulcanization to boost the palm oil industry. Polym. Polym. Compos..

[22] Kanadasan J., Razak H.A., Subramaniam V. (2018). Properties of high flowable mortar containing high volume palm oil clinker (POC) fine for eco-friendly construction. J. Clean. Prod..

[23] Adesina A., Awoyera P., Olalusi O.B. (2020). Agricultural wastes in concrete: Potential of oil palm kernel shell aggregate for lightweight concrete production. J. Solid Waste Technol. Manag..

[24] Amran M., Murali G., Fediuk R., Vatin N., Vasilev Y., Abdelgader H. (2021). Palm oil fuel ash-based eco-efficient concrete: A critical review of the short-term properties. Materials.

[25] Mohammed B.S., Foo W., Abdullahi M. (2014). Flexural strength of palm oil clinker concrete beams. Mater. Des..

[26] Shafigh P., Mahmud H., Jumaat M.Z. (2011). Effect of steel fiber on the mechanical properties of oil palm shell lightweight concrete. Mater. Des..

[27] Medeiros A., Zhang X., Ruiz G., Rena C.Y., Velasco M.d.S.L. (2015). Effect of the loading frequency on the compressive fatigue behavior of plain and fiber reinforced concrete. Int. J. Fatigue.

[28] Teo D.C., Mannan M.A., Kurian J.V. (2006). Flexural behaviour of reinforced lightweight concrete beams made with oil palm shell (OPS). J. Adv. Concr. Technol..

[29] MacGregor J.G., Wight J.K., Teng S., Irawan P. (1997). Reinforced Concrete: Mechanics and Design.

[30] Berhad T.C. Ordinary Portland Cement. http://www.tasekcement.com/index/products.html.

[31] ASTM (2020). Standard Practice for Making and Curing Concrete Test Specimens in the Laboratory.

[32] Hosen M.A., Shammas M.I., Shill S.K., Jumaat M.Z., Alengaram U.J., Ahmmad R., Althoey F., Islam A.S., Lin Y. (2021). Investigation of structural characteristics of palm oil clinker based high-strength lightweight concrete comprising steel fibers. J. Mater. Res. Technol..

[33] Malaysia S. Sika® ViscoCrete®-2199 High Range Water-Reducing Admixture for Early Strength Concrete. https://mys.sika.com/en/construction/concrete-admixtures/ready-mix-concrete/superplasticizers/sika-viscocrete-2199.html.

[34] Concrete S.F.F. Glued Hooked End Steel Fiber. https://www.cnsteelfiber.com/products/glued-hooked-end-steel-fiber/.

[35] BSI (2019). B.S.I. Testing Hardened Concrete Compressive Strength of Test Specimens.

[36] BSI (2019). B.S.I. Testing Hardened Concrete Flexural Strength of Test Specimens.

[37] ASTM International (2014). Standard Test Method for Static Modulus of Elasticity and Poisson’s Ratio of Concrete in Compression.

[38] Lo T.Y., Tang W., Cui H. (2007). The effects of aggregate properties on lightweight concrete. Build. Environ..

[39] Ahmmad R., Alengaram U.J., Jumaat M.Z., Sulong N.R., Yusuf M.O., Rehman M.A. (2017). Feasibility study on the use of high volume palm oil clinker waste in environmental friendly lightweight concrete. Constr. Build. Mater..

[40] Gao J., Sun W., Morino K. (1997). Mechanical properties of steel fiber-reinforced, high-strength, lightweight concrete. Cem. Concr. Compos..

[41] Shi C., Wu Y., Riefler M. (2005). Properties of fiber-reinforced lightweight concrete. ACI Spec. Publ..

[42] ASTM International (2017). Standard Specification for Lightweight Aggregates for Structural Concrete.

[43] Yazıcı Ş., İnan G., Tabak V. (2007). Effect of aspect ratio and volume fraction of steel fiber on the mechanical properties of SFRC. Constr. Build. Mater..

[44] Al-Rawi S., Taysi N. (2018). Performance of self-compacting geopolymer concrete with and without GGBFS and steel fiber. Adv. Concr. Constr..

[45] Balendran R., Zhou F., Nadeem A., Leung A. (2002). Influence of steel fibres on strength and ductility of normal and lightweight high strength concrete. Build. Environ..

[46] Düzgün O.A., Gül R., Aydin A.C. (2005). Effect of steel fibers on the mechanical properties of natural lightweight aggregate concrete. Mater. Lett..

[47] ACI Committee (2019). ACI 318-19: Building Code Requirements for Structural Concrete and Commentary.

[48] Cook C. (1983). FIP Manual of Lightweight Aggregate Concrete.

[49] Hamad B.S., Abou Haidar E.Y. (2011). Effect of steel fibers on bond strength of hooked bars in high-strength concrete. J. Mater. Civ. Eng..

[50] Hosen M.A., Alengaram U.J., Jumaat M.Z., Sulong N., Darain K. (2017). Glass fiber reinforced polymer (GFRP) bars for enhancing the flexural performance of RC beams using side-NSM technique. Polymers.

[51] Hosen M.A., Jumaat M.Z., Alengaram U.J., Sulong N.R. (2018). CFRP strips for enhancing flexural performance of RC beams by SNSM strengthening technique. Constr. Build. Mater..

[52] Ahmmad R., Jumaat M.Z., Alengaram U.J., Bahri S., Rehman M.A., bin Hashim H. (2016). Performance evaluation of palm oil clinker as coarse aggregate in high strength lightweight concrete. J. Clean. Prod..

[53] Qeshta I.M., Shafigh P., Jumaat M.Z., Abdulla A.I., Ibrahim Z., Alengaram U.J. (2014). The use of wire mesh–epoxy composite for enhancing the flexural performance of concrete beams. Mater. Des..

[54] Hosen M.A., Jumaat M.Z., Saiful Islam A., Obaydullah M., Darain M., Huda N. (2016). Investigation on energy absorption capacity of reinforced concrete beams by the near-surface mounted technique using ductile materials. Sci. Adv. Mater..

[55] Ahmmad R., Jumaat M., Bahri S., Islam A.S. (2014). Ductility performance of lightweight concrete element containing massive palm shell clinker. Constr. Build. Mater..

[56] Yap S.P., Bu C.H., Alengaram U.J., Mo K.H., Jumaat M.Z. (2014). Flexural toughness characteristics of steel–polypropylene hybrid fibre-reinforced oil palm shell concrete. Mater. Des..

[57] Huda M.N., Jumat M.Z.B., Islam A.S. (2016). Flexural performance of reinforced oil palm shell & palm oil clinker concrete (PSCC) beam. Constr. Build. Mater..

[58] Hosen M.A., Jumaat M.Z., Alengaram U.J., Sulong N.R., Alsubari B. (2019). Sustainable palm oil fuel ash mortar used as partial adhesive replacement in flexurally strengthened RC beams. J. Compos. Constr..

[59] Hosen M.A., Jumaat M.Z., Islam A.S. (2015). Side Near Surface Mounted (SNSM) technique for flexural enhancement of RC beams. Mater. Des..

[60] Thomsen H., Spacone E., Limkatanyu S., Camata G. (2004). Failure mode analyses of reinforced concrete beams strengthened in flexure with externally bonded fiber-reinforced polymers. J. Compos. Constr..

[61] Oudah F., El-Hacha R. (2012). A new ductility model of reinforced concrete beams strengthened using fiber reinforced polymer reinforcement. Compos. Part B Eng..

[62] Obaydullah M., Jumaat M.Z., Alengaram U.J., ud Darain K.M., Huda M.N., Hosen M.A. (2016). Prestressing of NSM steel strands to enhance the structural performance of prestressed concrete beams. Constr. Build. Mater..

